# A microexplosive shockwave-based drug delivery microsystem for treating hard-to-reach areas in the human body

**DOI:** 10.1038/s41378-022-00441-8

**Published:** 2022-09-23

**Authors:** Yi Sun, Wenzhong Lou, Hengzhen Feng, Wenting Su, Sining Lv

**Affiliations:** 1 Science and Technology on Electromechanical Dynamic Control Laboratory, School of Mechatronical Engineering, Beijing Institute of technology, Beijing, China; 2 Beijing Institute of Technology Chongqing Innovation Center, Chongqing, China

**Keywords:** Electrical and electronic engineering, Nanoscale materials, Nanoscale devices

## Abstract

Implantable drug-delivery microsystems have the capacity to locally meet therapeutic requirements by maximizing local drug efficacy and minimizing potential side effects. The internal organs of the human body including the esophagus, gastrointestinal tract, and respiratory tract, with anfractuos contours, all manifest with endoluminal lesions often located in a curved or zigzag area. The ability of localized drug delivery for these organs using existing therapeutic modalities is limited. Spraying a drug onto these areas and using the adhesion and water absorption properties of the drug powder to attach to lesion areas can provide effective treatment. This study aimed to report the development and application of microsystems based on microshockwave delivery of drugs. The devices comprised a warhead-like shell with a powder placed at the head of the device and a flexible rod that could be inserted at the tail. These devices had the capacity to deposit drugs on mucous membranes in curved or zigzag areas of organs in the body. The explosive impact characteristics of the device during drug delivery were analyzed by numerical simulation. In the experiment of drug delivery in pig intestines, we described the biosafety and drug delivery capacity of the system. We anticipate that such microsystems could be applied to a range of endoluminal diseases in curved or zigzag regions of the human body while maximizing the on-target effects of drugs.

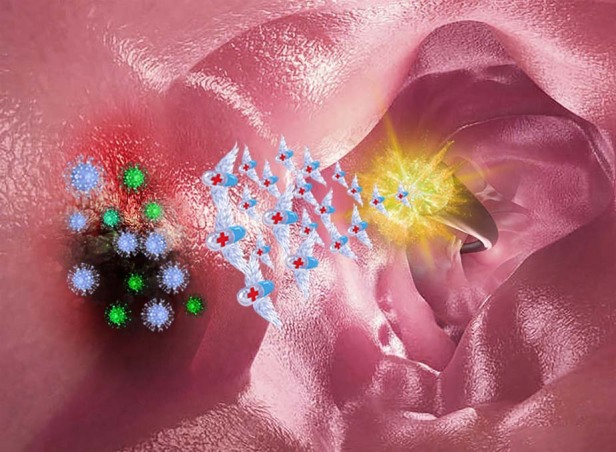

## Introduction

Microscale drug-delivery devices have recently drawn great attention due to their advantages over hypodermic needles, such as liquid jet injectors, powder injectors, microneedles, and thermal microablation, which help in tackling pain and needle phobia^1–4^. Implantable drug delivery devices have been applied for decades across a range of sites in the body, including the brain^5^. Endoluminal drug delivery systems, such as drug-eluting stents and drug-coated balloons, are effective at providing high local concentrations of antiproliferative agents and reducing vascular renarrowing (or restenosis) in vessels affected by obstructive atherosclerosis^6,7^. However, existing drug-coated balloons are limited in efficacy due to the limited transfer and retention of drugs from the balloon to the vessel wall. In the gastrointestinal tract, endoscopic injection, initially pioneered through the development of endoscopy-guided injection needles^8^, transformed the capacity to locally deliver therapeutics for a range of indications, including hemostasis with epinephrine^9^, sclerosant injection for variceal ablation^10^, submucosal lifts with normal saline and other materials^11^, and steroid injections for inflammation control and injection of biologics for inflammatory stricture management^12,13^. All these applications are aimed at a single point of treatment and do not solve the problem of regional lesion treatment. Babaee et al. at MIT developed a kirigami-based stent platform with the capacity to deposit drug depots circumferentially and longitudinally in the tubular mucosa of the gastrointestinal tract across millimeter to multicentimeter length scales, as well as in the vasculature and large airways^14^. However, since the device is made of hard silicone and is designed to be 8 cm in length and 12.5 mm in diameter, many lesions in the body occur in hard-to-reach areas, such as curved or zigzag areas of the gastrointestinal tract, trachea, or brain, to which conventional implantable drug delivery devices have difficulty delivering drugs. At the same time, the device used the internal air pressure change to make the surface microneedles pierce the human tissue to release the drug, and the single-time drug release speed is slow, which is likely to cause pain and discomfort. In addition, Zaizai et al. presented a simple 3D electroporation platform that enables massively parallel single-cell manipulation and the intracellular delivery of macromolecules and small molecules^15^, which provides a simple, efficient, high-throughput intracellular delivery method that may facilitate on-chip cell manipulation, intracellular investigation and cancer therapy. Long et al. proposed a multimicrochannel microneedle microporation (4 M) platform that achieves high efficiency, safety, and uniformity for in vivo intracellular delivery^16^. This platform has proven to be efficient for the delivery of chemotherapeutics in solid tumors in vitro and in vivo, with significantly enhanced anticancer effects and reduced systemic toxicity, and serves as a general-purpose delivery tool for emerging drugs in vivo.

The principle of jet injection has been used for drug delivery for a long time^17^, allowing drugs to be sprayed through a narrow orifice to a lesion or inflammatory area. Currently available commercial devices employ a variety of forms of stored energy, including compressed springs, compressed gases, explosive chemicals, Lorentz forces, electric pulse microjets, and shockwaves. Electrically pulsed microjet piezoelectric actuators have been used to deliver injections, albeit to restricted tissue depths, and are mainly applied to the body surface^18^. Taberner et al. developed a controllable injection device^19^, which operated by ejecting a liquid drug through a narrow orifice at high pressure, thereby creating a fine high-speed fluid jet that could readily penetrate skin and tissue. However, the device needed a linear power amplifier and a peak power of 4 kW to drive the Lorentz force motor to work. The complex system and large volume make it difficult to use in the targeted therapy of diseases in vivo. Shockwaves are one of the most efficient mechanisms of energy dissipation observed in nature. Sudden energy release (within a few microseconds) leads to the formation of shockwaves. Shockwaves have been used successfully for disintegrating kidney stones^20^, noninvasive angiogenic therapy^21^, and osteoporosis treatment^22^. Rathod et al. introduced a microshock tube-based drug delivery device^23^; however, the entire inner wall of the tube of the device was coated with an explosive coating, and in the process of generating an axial shockwave, there was also radial impact to cause the tube wall to expand. The use of aluminum and copper films in the device results in poor biocompatibility. Tagawa et al. managed to generate thin, focused microjets to model the penetration of the jet into soft matter and human skin enclosing soft tissue^24^ and solved the problem of the diffusion of microjets during the needle-free injection. However, the inability of this device to deliver drugs to the human body limits treatment options. Jagadeesh et al. proposed a typical needleless drug delivery device consisting of a diaphragm and an explosive driver to propel the liquid drug to the target^25^. However, the study did not report device calibration considering drug particle and skin properties. Several typical targeted drug delivery microsystems were compared with our study. The detailed results are shown in Supplementary Table S1.

In this study, we reported a microsystem for drug delivery based on microexplosive shockwave injection. This warhead-like device, with an outer diameter ranging from a few millimeters to a centimeter and a flexible rod that could be inserted at the tail, can deliver drugs deep into curved or zigzag areas of the body. Highly doped semiconductor bridges are used for energetic devices that generate high energy for a short time under the application of a pulse current of a minimum of 1 A and apply energy to the energetic material copper azide. Copper azide receives heat and reacts chemically, and then explodes and produces shockwaves. The powder at the head of the device is sprayed into the pathological area of the human body due to the shockwave generated by the explosion. Under the application of the shockwave, the drug powder in the transparent cover obtainded a maximum kinetic energy of 3.2 × 10^−4^ J, and the maximum speed reached 60 m/s. In addition, due to the internal structure design of the system, the microexplosive shockwave was mainly transmitted in the axial direction, and the radial vibration was extremely small. A PDMS film is set in the tubular shell so that the copper compounds generated by the explosion are trapped in the shell and do not enter the human body with the powder. The overall operating time of the system is only a few hundred microseconds. Thus, heat generation is negligible, as the device does not heat up. We designed the system and each module, including the energetic material copper azide and the semiconductor bridge as the energetic device.

Then, we performed a dynamic simulation evaluation of the influence of geometric characteristic parameters on the drug injection of the explosive shockwave, as well as the processing and assembly of the system and each module. Finally, experimental tests were carried out in pig intestines, and the results were analyzed to verify the biosafety and drug delivery capacity of the microexplosive shockwave microsystem.

## Working mechanism

### Schematic and structure design of the drug release microsystem

**Fig. 1:**
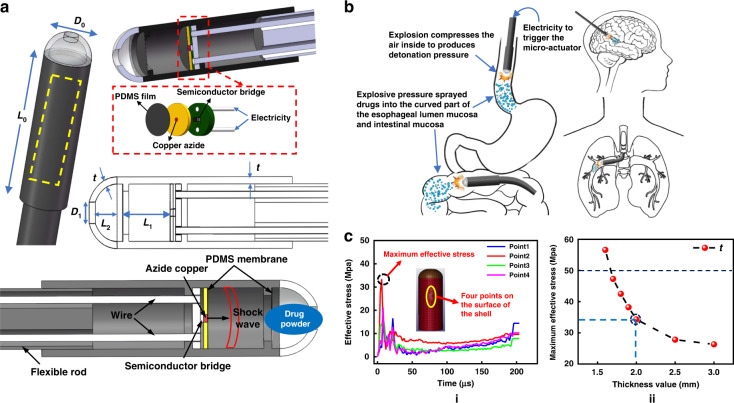
Overview and numerical characterization of drug delivery microsystem based on microexplosion shockwave injection. **a** Schematic diagram of the structure and characteristic dimensions of the explosive shockwave-based drug delivery microsystem. **b** An implantable targeted drug delivery microsystem performs shockwave drug injection in the gastrointestinal tract, brain, and curved or flexed area of blood vessels. **c** Variation in effective stress at different points on the shell under shockwave and the relation between the thickness *t* of the tubular shell and the maximum stress. The red circle indicates the final manufacturing selection of the shell thickness value

Figure 1a shows a schematic diagram of a warhead-like microexplosive shockwave-based drug delivery microsystem. The device consisted of a transparent cover on the head, a tubular shell in the middle, and a flexible rod at the tail. Two steps were set inside the tubular shell, and the PCB was welded with a semiconductor bridge. The energetic material copper azide and the PDMS film were closely attached to form an energetic system with a sandwich structure and fixed on the back step to generate shockwaves. The solder holes on the PCB directed the metal wires out, supplying power outside the system through the holes of the flexible rod. A layer of PDMS film was also fixed on the first step of the head of the tubular shell near the transparent cover. The main function of the two PDMS films inside the shell was to attenuate the internal shockwave and prevent byproducts after the reaction of copper azide entering the biological body together with drugs in the transparent cover. The distance between the two PDMS films was *L*_1_, the thickness of the shell and the transparent cover were both *t*, and the top of the transparent cover was provided with a circular hole with a diameter of *D*_**1**_ through which the drug powder was sprayed. This microsystem could be manufactured in different sizes and used for drug delivery deep into the gastrointestinal tract, brain, and blood vessels under an endoscope. Figure 1b shows that the device was located in curved areas such as the esophagus, small intestine, blood vessels, and brain to deliver drugs. The drug could be attached to the lesion through its adhesiveness and water absorption to provide effective treatment.

The tubular shell and the transparent cover were made of ASTM resin, which is a biocompatible resin material, with a maximum breaking strength of 65 MPa. The unsafe strength area of the shell fracture is between 50 MPa and 65 MPa, and the material parameters are shown in Supplementary Fig. S2.

We implemented the finite element analysis of the explosion impact in LS-DYNA and selected four points on the shell surface. Since we hoped to obtain the maximum effective stress on the shell surface when the shockwave occurred, all four selected points were on the shell surface near the energetic material area. Figure 1c(i) shows the effective stress of four points on the shell under the internal shockwave when the shell thickness *t* was 2 mm. The maximum effective stress was 38.7 MPa, which did not exceed 50 MPa. Figure 1c(ii) shows the change in the maximum effective stress on the outer surface of the shell with the thickness *t* of the tubular shell and the transparent cover from 3 mm to 1.6 mm. The value of *t* decreased from 3 mm to 1.6 mm, and the maximum effective stress increased; when the value of *t* was between 2 mm and 1.6 mm, the maximum effective stress increased faster. When the value of *t* was 1.6 mm, the maximum effective stress exceeded 50 MPa, and when the value of *t* was 1.5 mm, the shell fractured under shockwave finite element simulation. Research has shown that when the shell thickness *t* is 2 mm or more than 2 mm, the system worked reliably. However, choosing a larger value of *t* resulted in a smaller internal space and increased the difficulty of manufacturing and assembly. Therefore, in the case of the outer diameter of the shell *D*_0_ = 10 mm, we chose the thickness *t* of the shell as the ideal value. After determining the thickness *t* of the shell and transparent cover, we continue to design, manufacture and evaluate the performance of the internal energetic system.

### Performance characterization of the copper azide

**Fig. 2:**
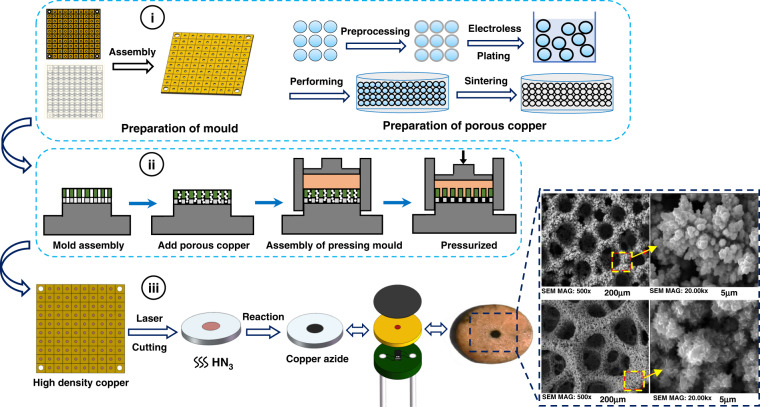
Preparation process of copper azide. i Preparation of the energetic material mold and nanoporous copper. ii Preparation of the high-density porous copper powder: mold assembly, adding porous copper powder, adding a pressing mold, and finally pressing to obtain high-density porous copper. iii Ammoniating high-density porous copper to obtain copper azide, the crystal phase diagram of porous copper and copper azide under the electron microscope

In the drug delivery microsystem, copper azide was used as an energetic material to generate an explosive shockwave, which was characterized by a small detonation wavefront area, high-energy density, and a deflagration point of 195 °C. The copper azide used in this study was light in weight and small in volume. The copper azide we prepared was 300 μm in thickness and 1 mm in diameter due to factors such as process technology. Figure 2 shows the process flow for preparing copper azide. As shown in Fig. 2(i), the mold and the nanoporous copper needed for copper azide were designed and prepared; the nanoporous copper was prepared by the polystyrene template method. The polystyrene spheres (PS)/Cu core–shell structure was prepared by depositing a layer of copper on the surface of polystyrene microspheres by electroless plating, and then the PS/Cu core–shell structure was obtained by grinding and sintering the microspheres to obtain nanoporous copper with a large pore structure. As shown in Fig. 2(ii), the mold plate was first assembled, and nanoporous copper powder was added. Then, the pressing mold was added, and finally, the high-density copper powder was obtained by pressure. As shown in Fig. 2(iii), high-density porous copper and the mold were laser-cut, and the nanoporous copper was ammoniated to obtain high-density copper azide. In addition, we could observe the crystal phase diagram of porous copper and copper azide under an electron microscope.

### Structure and performance characterization of the semiconductor bridge

**Fig. 3:**
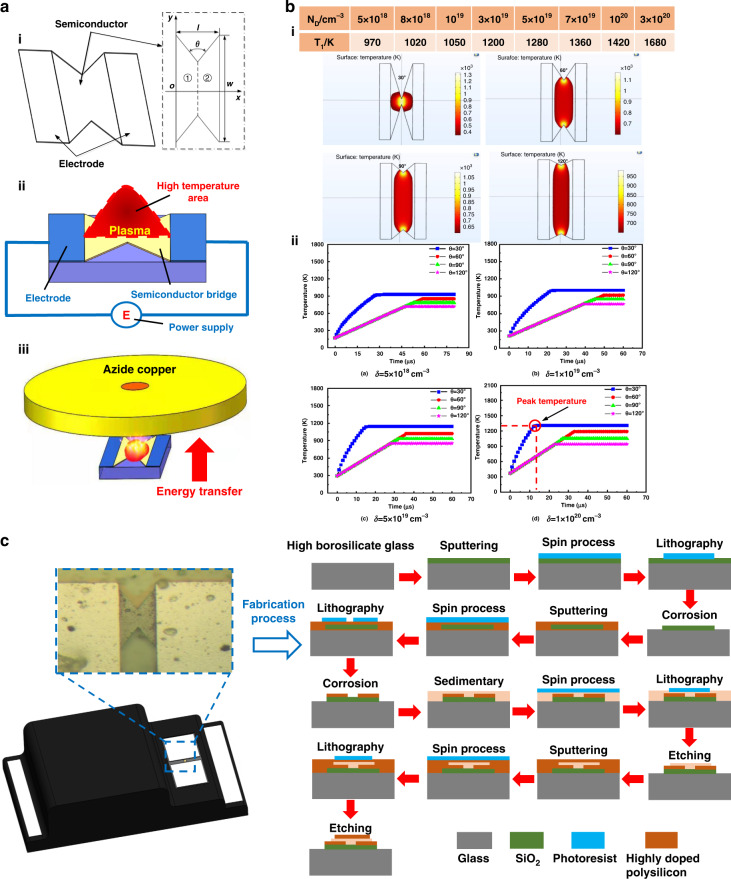
Semiconductor bridge design, temperature field simulation, and processing. **a** Structure design, energy generation, and application mechanism of a double V-type semiconductor bridge. **b** Temperature field simulation of the semiconductor bridge with different V-shaped angles *θ* and different doping concentrations δ. **c** Semiconductor bridge process flow

The microsemiconductor bridge provided an energy source for the shockwave-based drug delivery microsystem, and its structure and material properties had an essential influence on the full release of the energy of the energetic drug copper azide. As shown in Fig. 3a-ii, the pulse current flowed through the bridge area, and the current density was the highest at the gap, forming a burst point. The semiconductor bridge vaporized rapidly under Joule heating and formed a weak plasma discharge under the application of an electric field; the bridge area reached more than 1000 K in a very short time. When the semiconductor bridge was vaporized, the circuit was disconnected and the temperature did not continue to rise at this time. As shown in Fig. 3a-iii, the semiconductor bridge provided ignition energy to the copper azide. After heat conduction, the copper azide chemically reacted and continued to generate heat. Finally, an explosion occurred, and a shockwave was generated. In the process of ignition energy transfer from the semiconductor bridge to copper azide, at higher peak temperatures and faster heat conduction, the heat received by copper azide was greater than the heat dissipated, and the shockwave generated by the explosion was stronger. When the heat generation rate or the peak temperature of the semiconductor bridge decreased, it was difficult for the energetic material copper azide to ignite and then detonate. A picture of the semiconductor bridge and an enlarged view of the bridge area under the electron microscope are shown in Supplementary Fig. S4.

The peak temperature and temperature rise rate of the semiconductor bridge were related to the resistivity of the semiconductor bridge. The resistivity of the semiconductor bridge was determined by the aspect ratio *ω*, the V-shaped angle *θ*, and the doping concentration of nonmetallic elements, that is, *R* = *R*(*ω*, ND, *θ*, *ρ*). The length direction of the semiconductor bridge was taken as the *x* axis, and the width direction was taken as the *y* axis to establish a rectangular coordinate system, as shown in Fig. 3a-i. The resistance was obtained by integrating the two parts of ① and ②, and the cross-sectional area of the semiconductor bridge area along the *x*-axis could be expressed as:

1 $$S(x) = \left\{ {\begin{array}{*{20}{l}} {\delta \left( {w - 2xctg\frac{\theta }{2}} \right)} \hfill & {0 \le x \le \frac{1}{2}} \hfill \\ {\delta \left[ {w - 2(l - x)ctg\frac{\theta }{2}} \right]} \hfill & {\frac{1}{2} \le x \le l} \hfill \end{array}} \right.$$

For a conductor with a constant cross-section, the resistance was proportional to the length ***l*** and inversely proportional to the cross-section *S*, and the resistance was calculated as follows:

2 $$R = \rho \frac{l}{S}$$

The integral and sum of the resistance at 0 ≤ *x* ≤ *l/*2 and *l*/2 ≤ *x* ≤ *l* yielded:

3 $$R = \frac{\rho }{\delta }\frac{{l[2w - lctg(\theta /2)]}}{{2w[w - lctg(\theta /2)]}}$$

where *ρ*/*δ* = *R*_s_ is the square resistance of the semiconductor bridge. Let *ω* = *l*/*w*, known as the aspect ratio, then:

4 $$R = \frac{{R_s\omega }}{2}\left[ {1 + \frac{1}{{1 - \omega ctg(\theta /2)}}} \right]$$

Equation (3) is the theoretical calculation formula for the resistance of the V-shaped semiconductor bridge. When the square resistance was determined, the resistance of the semiconductor bridge was only related to the aspect ratio *ω* and the V-shaped angle *θ*. The aspect ratio of the semiconductor bridge was generally approximately 1:4, and the typical size was 100 μm × 400 μm × 2 μm. The design of the V-shaped angle *θ* needed to meet a certain range, that is, 2argtan*ω* < *θ* < π; when *θ* = π, the *R* value was the smallest; when *θ* = 2argtan*ω*, since *ω* = *l*/*w* = 0.25, *θ* = 2argtan*ω* = 28°; at this time, *R* was infinite. In addition to the semiconductor bridge structure, knowing the change in square resistance with temperature was also necessary. Since the doping thickness was generally *δ* = 2 μm, it was necessary to calculate the change in resistivity with temperature.

According to the principle of semiconductor physics and the calculation method of a double V-shaped semiconductor bridge^26^, the expressions of the resistivity of the semiconductor bridge in different temperature regions can be obtained:

5 $$\rho = \left\{ {\begin{array}{*{20}{l}} {\frac{1}{{N_Dq\mu _n}}} \hfill & {T_0 \le T \le T_1} \hfill \\ {\frac{1}{{(N_D \,+\, n_i^2/N_D)q\mu _n}}} \hfill & {T_1 \le T \le T_{melt}} \hfill \\ {\frac{1}{{\rho (T_{melt}) \,-\, 2.7(t \,-\, T_{melt})}}} \hfill & {T_{melt} \le T \le T_g} \hfill \end{array}} \right.$$

In Eq. (5), *T*_*g*_ = 2880 K and *T*_melt_ = 1684 K are the boiling point and melting point of the semiconductor bridge, respectively; *T*_*1*_ is the upper limit of the saturation ionization temperature; and *T*_0_ is the normal temperature of 300 K. The relationship between the doping concentration and the upper limit of the saturated ionization temperature *T*_1_ is shown in Fig. 3b-i.

We analyzed the temperature field of the semiconductor bridge with different V-shaped angles in the COMSOL finite element software. Figure 3b-ii shows that when the V-shaped angle of the semiconductor bridge was 30°, the peak temperature was higher, and the energy was more concentrated. As the V-shaped angle *θ* increased, the peak temperature was smaller and the temperature field was more divergent. Additionally, the temperature field variation with time of the semiconductor bridge with different V-shaped angles was studied under different doping concentrations of nonmetallic elements, as shown in Fig. 3b-ii. The higher the doping concentration was, the higher the peak temperature was, and the faster the peak temperature was reached. We also experimentally verified that the semiconductor bridge could successfully initiate copper azide. The structure and chemical parameters of the semiconductor bridge were determined based on finite element analysis and experiments. The manufacturing process is shown in Fig. 3c:

1. Silicon wafer cleaning and preparation
2. Magnetron sputtering silicon oxide, with a thickness of 500 nm
3. Spin-coated photoresist, with a thickness of 4 μm
4. Lithography used to select the location of the semiconductor bridge area
5. Removal of photoresist and wet etching of silicon oxide with hydrofluoric acid
6. Sputtering highly doped silicon, with a thickness of 2 μm
7. Spin coating photoresist, glue process, the thickness of 4 μm
8. Lithography used to select the semiconductor bridge area graphically
9. TMAH wet corrosion process to complete the release of the silicon electrode structure
10. Metal electrode deposition, Al, with a thickness of 200 nm
11. Spin coating photoresist, with a thickness of 400 nm
12. Forming a metal electrode in the semiconductor bridge area
13. Dry etching (ICP) metal electrode
14. Sputtering highly doped silicon with a thickness of 2 μm
15. Spin-coated photoresist, with a thickness of 400 nm
16. Lithography used to graphically select the semiconductor bridge area
17. Fabrication of the semiconductor bridge area by a dry etching process (ICP).

## Results and discussion

### Performance of the microshockwave in drug release

**Fig. 4:**
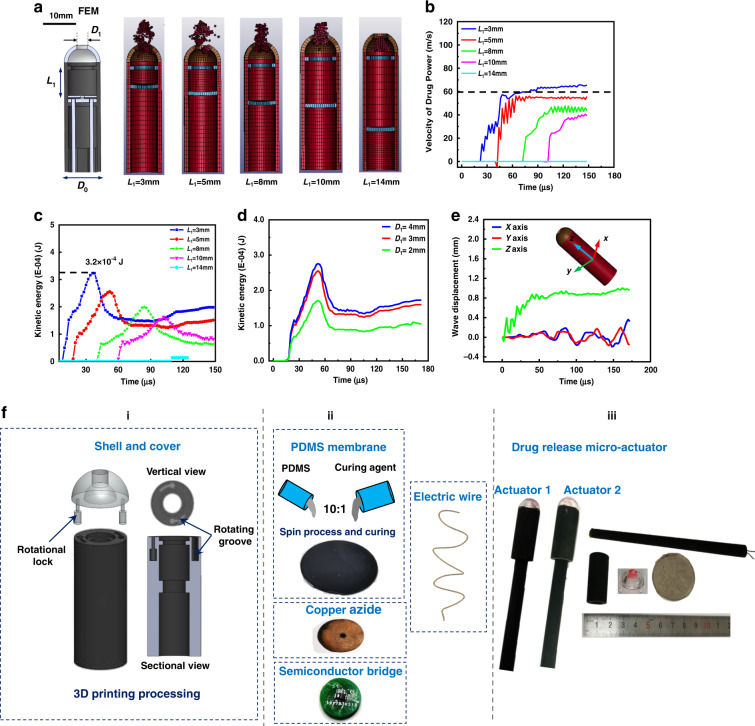
The characteristics of drug delivery based on microexplosive shockwaves. **a** The effect of drug injection by shockwave under different L1 values. **b** Under the condition of different L1 values, the relationship between the injection velocity and time of a single particle of powder under the application of the shockwave. **c** Under different L1 values, the change in kinetic energy of the drug powder under the application of the shockwave. **d** Effect of the hole diameter D1 of the transparent cover on the kinetic energy of drug powder injection under the shockwave. **e** The vibration displacement of the microsystem in the *x*, *y*, and *z* directions during the shockwave. **f** The manufacturing process of the implantable shockwave-based drug delivery microsystem: (i) 3D printing and processing of the tubular shell, transparent cover, and flexible push rod, (ii) preparation of PDMS film, copper azide, circuit module and semiconductor bridge, and (iii)) microsystem assembly

Since the drug spray under the microexplosive shockwave was completed in the order of microseconds, we needed to evaluate the important dimensional parameters in the finite element model. As shown in Fig. 4a, we reported the ability of the drug powder to spray under the microexplosive shockwave at different *L*_1_ values. The amount of drug powder sprayed decreased with increasing *L*_1_ value; when *L*_1_ = 14 mm, no powder was sprayed. As shown in Fig. 4b, c, we used the velocity of the top single particle in the powder and the kinetic energy of the all powder over time in the finite element analysis. An increase in the value of *L*_1_ caused a decrease in the speed of the powder particles. The kinetic energy first increased and then decreased. This was because the kinetic energy of the powder decreased when the powder passed through the hole of diameter *D*_1_ and then stabilized. Considering the assembly methods and the integration relationship between the components, we believed that an *L*_1_ value of 5 mm or 8 mm was more appropriate. In the case of *L*_1_ = 5 mm, we analyzed the influence of the diameter *D*_1_ of the hole on the transparent cover on the drug powder spray. We found that the kinetic energy decreased significantly when the hole diameter was less than 3 mm. Interestingly, when the hole diameter was larger than 3 mm, the kinetic energy of powder injection did not increase significantly; a hole diameter that was too large reduced the amount of drug powder loaded. Considering the size of the outer diameter of the tubular shell *D*_0_ = 10 mm, we believed that 3 mm *D*_1_ was the most ideal choice. During the generation of shockwaves by the microsystem to release the drug. The vibration displacement of the system shell in the *x*, *y*, and *z* directions is shown in Fig. 4e. The vibration displacement in the *x* and *y* directions was within +0.4 mm and −0.4 mm, respectively, and the vibration displacement in the *z* direction was within 1 mm. Since the working area of the drug delivery microsystem in the body was mostly in the range of several millimeters to several centimeters, the vibration at this value could be ignored.

A visualized schematic diagram of the manufacturing process of the targeted drug delivery microsystem is shown in Fig. 4f. The tubular shell, transparent cover, and flexible rod of the system were processed by 3D printing. The shell and transparent cover were made of ASTM resin, and the push rod was made of rubber. The tubular shell was provided with a rotating groove, and correspondingly, a protruding rotating buckle was provided on the transparent cover. As shown in Fig. 4f-i, the rotating buckle was in the shape of an inverted hammer. The rotating buckle was inserted into the rotating groove and then rotated counterclockwise; the transparent cover, and the tubular rotating shell could be fixed axially. The PDMS and the curing agent were mixed in a ratio of 10:1. The mixed liquid glue was dropped on the homogenizer and spin-spread. The curing was accelerated in a 100 °C incubator to form the 1 mm-thick PDMS film. we needed. The PDMS film with a diameter of 8 mm was cut. The tubular housing, transparent cover, copper azide, circuit module welded with the semiconductor bridge, PDMS film, and metal wire were assembled. The assembled drug delivery microsystem is shown in Fig. 4f-iii. The flexible rod was designed into a conical tube to facilitate assembly and fixation. We assembled two devices with sizes ***L***_**1**_ of 5 mm and 8 mm.

### Evaluation of the drug delivery effect of the microsystem

**Fig. 5:**
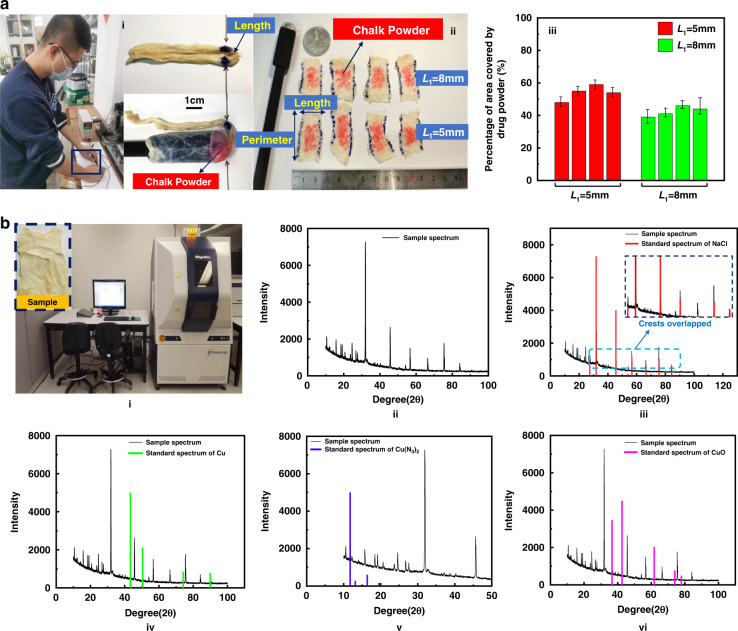
The feasibility and biosafety of the design are verified by pig intestine drug delivery experiments. **a** Test results of the feasibility of the design. **b** Multifunctional X-ray diffractometer scans the sample and compares it with the standard spectrum of the components to be detected. ii–vi Test result data

We used pig intestines to conduct drug injection experiments to evaluate the feasibility and biological safety of the drug delivery microsystem based on explosive shockwave injection. The pig intestines were folded in half to create a curve, and the curved section on the pig intestines was marked with a marker pen. The curve area is shown in Fig. 5a-i. Our goal was to deliver the drug powder to the marked area by means of shockwave injection. Red chalk powder was used to highlight the experimental effect. The device was inserted into the pig’s intestine, the top of the transparent cover was aligned with one of the marks, and the pig’s intestine was always in a curved state during the entire experiment. The external power supply was 5 V, and the experiment was started. According to the two types of *L*_1_ value microsystems provided in the previous section, four sets of experiments were performed for each of the two types of microsystems. The pig intestines were cut after the test along the axial direction. The result is shown in Fig. 5a-ii. The red chalk powder was sprayed into the intestines and occupied up to 60% of the local area, proving the feasibility of the design. As shown in Fig. 5a-iii, when the *L*_1_ value of the microsystem was small, the powder area sprayed on the pig intestine was large, and the color was darker in comparison. This was because in this case, the shockwave was stronger.

Since copper azide produced copper and nitrogen after explosion^27^, copper could cause heavy metal poisoning in the human body. Therefore, we needed to carry out biological safety experiments in pig intestines, and used salt as drug powder. After the experiment was completed, we dried the sample and performed phase detection under a multifunctional X-ray diffractometer (Rigaku SmartLab, Japan), as shown in Fig. 5b. Figure 5b-ii is the sample spectrum after scanning; the sample spectrum was compared with the standard spectrum of the components to be detected. The peak intensity of the sample spectrum was completely consistent with the standard spectrum of NaCl, which proved that the sample contained the powder sprayed by the microsystem, as shown in Fig. 5b-iii. We could also see that the sample spectrum had no peak intensity at the standard spectra of Cu, Cu(N_3_)_2_ and CuO, which proved that no Cu, Cu(N_3_)_2_, or CuO were sprayed onto the pig intestine with the drug powder.

## Conclusion

In summary, given the recognized capacity of implantable targeted therapy for disease treatment, we report a new type of biomedical device. The explosive shockwave not only sprayed the drug powder into the tubular area of the body but also, more importantly, delivered the drug powder to the hard-to-reach areas of the gastrointestinal tract, brain, trachea, and other organs in the body, such as the curved or zigzag areas. The use of drug adhesion and water absorption to attach the drug to the lesion provided effective treatment. In this study, the overall design of the drug delivery microsystem based on the microexplosive shockwave injection was carried out. The combination of finite element simulation and experiments was used to evaluate the characteristic parameters of the tubular shell, transparent cover, semiconductor bridge, and other modules and to analyze the influence of the characteristic parameters on shockwave drug delivery. Finally, the optimal parameters were selected to process modules of the microsystem. More importantly, we demonstrated the capacity of the microsystem for targeted drug delivery in our experiments. The maximum injection area reached more than 60% of the local area, and the system was nontoxic and free of collateral damage. In addition, the system could match different structural sizes according to the size of the human body cavity and was applied to treat a series of lesions in hard-to-reach areas in the body.

The main contribution and novelty of this work can be summarized as follows: (1) This work proposed a microexplosive shockwave-based drug delivery microsystem, which can realize parametric design and processing to meet the treatment of different areas in the body. The flexible rod of this device enables drug delivery in hard-to-reach areas such as curved or zigzag areas in the body. (2) In this study, a high-energy semiconductor bridge and a high-density explosive, copper azide, were designed and fabricated so that the system could release high energy in tens of microseconds driven by a small amount of electricity (a minimum electric current of 1 A). The copper azide was ignited, and an explosive shockwave was generated so that the drug powder could be sprayed onto the surface of the lesion at a maximum speed of 60 m/s in the human body. Due to the extremely short drug spraying time, this process will not cause pain to or heat the human body. In addition, the internal structure of the device was designed so that the explosive shockwave is transmitted mainly in the axial direction and the radial vibration is small, resulting in better safety. (3) The integrated microsystem was applied to pig intestines and other experimental tests to verify the drug delivery ability and biosafety.

## Materials and methods

### Microexplosive shockwave drug spray simulation

Finite element simulation of drug spray under microexplosive shockwave was carried out to evaluate the important parameters of microsystem structure. The instruction set modeling was conducted with Truegrid software, and the geometric parameters, material properties, constraints, and explosion boundary of this structure were mainly set. *L*_1_ = 3 mm was taken as an example (*L*_1_ is the distance between the two PDMS films, that is, the distance of the explosive shockwave in the tube). The specific steps of modeling are as follows: first, the center of the hemispherical transparent cover was set as the origin, from which the coordinate position of each device of the microsystem can be known. Combined with the index mapping method, the transparent cover, tubular shell, PDMS film, copper azide, and circuit board were modeled according to the instructions. The model was presented in the form of a grid, as shown in Supplementary Fig. S1. The explosive impact process follows the CJ theory of detonation wave, and the propagation velocity of detonation wave is *U*. On the wavefront, the relationship between mass conservation, momentum conservation, and energy conservation can be obtained:

6 $$m = \rho _0(U - v_0) = \rho (U - v)$$

7 $$\rho _0(U - v_0)^2 - \rho (U - v)^2 = p - p_0$$

8 $$m\left[ {e_0 + \frac{1}{2}\left( {U - v_0} \right)^2 - \frac{1}{2}\left( {U - v} \right)^2\,+\, Q_{\tilde v}} \right] = p_0v_0 - pv$$

In the formulas, *p*, *ρ*, *v*, and *e* represent the pressure, density, velocity, and specific internal energy in the explosion, and *p*_0_, *ρ*_0_, *v*_0_, *e*_0_ represent the initial state of the explosive pressure, density, velocity, specific internal energy, respectively. The particle velocity before detonation is *v*_0_ = 0, and *ρ* = 1/$\tilde v$ ($\tilde v$ is the specific volume). *ρ* = 1/$\tilde v$ was brought into Eqs. (6) and (7), and the sorted results were inserted into (8) to finally obtain:

9 $$p - p_0 = - \frac{{U^2}}{{\tilde v_0^2}}\tilde v + \frac{{U^2}}{{\tilde v_0}}$$

Then we calculated the constructed model in LS-DYNA. After the calculation was completed, the results were post-processed in LS-PrePost, and the relationship between the kinetic energy of the drug powder and time was obtained. Finally, by changing the parameters *L*_1_ and *D*_1_, we analyzed the effect of different *L*_1_ and *D*_1_ values on the kinetic energy of the powder.

### Development of an explosive shockwave-based drug delivery microsystem

The preparation of the microsystem was mainly divided into three steps: (1) design and processing of the warhead-like shell; (2) design and processing of the energetic device semiconductor bridge; and (3) preparation of the energetic material copper azide.

The microsystem mainly included a transparent cover, a tubular shell in the middle part, and a flexible rod at the tail. All three parts were processed by 3D printing (Xingyou Technology Co., Ltd., China).

Next, we proceeded to prepare a semiconductor bridge. The preparation process required using a lithography machine, a homogenizer, an etching machine and a PECVD coating machine (Institute of Semiconductors, Chinese Academy of Sciences, China). The preparation process started from the silicon wafer. First, the silicon wafer was prepared and cleaned, and 500-nm thick silicon oxide was sputtered on the silicon wafer. The wafer was spun on the photoresist, the position of the semiconductor bridge was selected, and then the photoresist was removed. Highly doped low resistance silicon with a thickness of 2 μm was sputtered on silicon oxide, and then the semiconductor bridge area was prepared by dry etching (ICP). The photoresist was spin-coated, and then photoetched. Then the silicon-based electrode structure was completed by the TMAH wet etching process, aluminum metal with a thickness of 200 nm was deposited, and finally the metal electrode was prepared by dry etching (ICP). Hence, the semiconductor bridge was prepared. The semiconductor bridge was a low-power device that generated high energy under a low-voltage drive. The drive voltage of this design was 5 V.

Then, copper azide was prepared, and copper sulfate (0.1 mol L^−1^) pentahydrate and ethylene diamine tetraacetic acid (0.12 mol L^−1^) were dissolved in deionized water. The sodium hydroxide solution was added slowly at a high stirring speed to a pH of 12.85. The preprocessed PS (0.2 g L^−1^) was dispersed into the alkaline bath, followed by the addition of formaldehyde (15 ml L^−1^) to induce a deposition reaction. The plating process was conducted at 40 °C for 15 min under ultrasound. PS/Cu core–shell microspheres were collected by centrifuging the mixed solution at 4000 rpm for 4 min, washed with deionized water three times, and dried under vacuum at 50 °C for 24 h to yield PS/Cu powders. Then, 0.1 g powders were pressed by a powder pressing machine to form a round film. An NPC film was produced by sintering the PS/Cu film with a heating rate of 5 °C/min in a N_2_ atmosphere at 400 °C for 1 h to remove PS templates. The NPC film was pressed into the pores in polycarbonate confinements, which were custom-made to fabricate a copper azide microcharge. Furthermore, 2 g sodium azide and 10 g stearic acid were mixed in a three-neck round-bottom flask. The left neck was equipped with a gas inlet valve, and a thermometer was placed on the right neck of the flask. The middle neck was connected to a custom glass-made sand core filter to place NPC-filled polycarbonate confinements separately for maximum exposure to HN_3_. The top of the filter was connected to a buffer absorption device with saturated KOH to filter unreacted HN_3_. Before the reaction, the left neck was introduced to N_2_ for 10 min to exclude O_2_ from the system and then the flask was heated with simethicone to 135 °C. After the reaction, N_2_ was also introduced for 10 min to absorb residual HN_3_. After every 12 h, 2 g sodium azide and 10 g stearic acid were added to the flask to confirm a steady stream of HN_3_ gas. Finally, copper azide was obtained.

### Experimental setup and measurement system

In the experiment to verify the feasibility of this design, fresh pig intestines were used. The red chalk powder was placed in the transparent cover of the microsystem, and the device was inserted into the pig intestines. After the test, the samples were cut along the axial direction, and the proportion of the red powder in the local area was observed and measured. In the biosafety test, salt was used as a drug powder to spray into pig intestines. After the experiment, we used a multifunctional X-ray diffractometer (Beijing Institute of Technology, China) to inspect the samples. We put the samples into the equipment and set the scanning angle from 10° to 90°. In addition, we performed drug ejection experiments on a heart model (3D printed in a 1:1 ratio); see Supplementary Fig. S3. Due to the flexible characteristics of the device, chalk powder was sprayed into the cardiac lumen of the heart through the blood vessels. After the experiment, the connecting part of the blood vessel and the heart was cut, and it was observed that the inner cavity was sprayed with red powder.

## Supplementary information

Animation display

Supplementary figure

## References

[1] Arora A, Prausnitz MR, Mitragotri S (2008). Micro-scale devices for transdermal drug delivery. Int. J. Pharm..

[2] Brown MB, Martin GP, Jones SA, Akomeah FK (2006). Dermal and transdermal drug delivery systems: current and future prospects. Drug Deliv..

[3] Jain KK (2008). Drug delivery systems—an overview. Methods Mol. Biol..

[4] Schramm J, Mitragotri S (2002). Transdermal drug delivery by jet injectors: energetics of jet formation and penetration. Pharm. Res..

[5] Brem H (1989). Biocompatibility of a biodegradable, controlled-release polymer in the rabbit brain. Sel. Cancer Ther..

[6] Kearney CJ, Mooney DJ (2013). Macroscale delivery systems for molecular and cellular payloads. Nat. Mater..

[7] Stefanini GG, Holmes DR (2013). Drug therapy: drug-eluting coronary-artery stents. N. Engl. J. Med..

[8] Nelson DB (1999). Technology status evaluation report: injection needles. Gastrointest. Endosc..

[9] Chung SS (1997). Randomised comparison between adrenaline injection alone and adrenaline injection plus heat probe treatment for actively bleeding ulcers. BMJ.

[10] Soehendra N, Bohnaker S, Binmoeller KF (1997). Nonvariceal upper gastrointestinal bleeding. N. Alternative Hemostatic Tech. Gastrointest. Endosc. Clin. North Am..

[11] Pang Y (2019). Endoscopically injectable shear-thinning hydrogels facilitating polyp removal. Adv. Sci..

[12] Usman RM, Jehangir Q, Bilal M (2019). Recurrent esophageal stricture secondary to pemphigus Vulgaris: a rare diagnostic and therapeutic challenge. ACG Case Rep. J..

[13] Swaminath A, Lichtiger S (2008). Dilation of colonic strictures by intralesional injection of infliximab in patients with Crohn’s colitis. Inflamm. Bowel Dis..

[14] Babaee S (2021). Kirigami-inspired stents for sustained local delivery of therapeutics. Nat. Mater..

[15] Zaizai D (2020). On-chip multiplexed single-cell patterning and controllable intracellular delivery. Microsyst. Nanoeng..

[16] Long, L. et al. Multimicrochannel microneedle microporation platform for enhanced intracellular drug delivery. *Adv. Funct. Mater*. **32**, 2109187 (2021).

[17] Mitragotri S (2006). Current status and future prospects of needle-free liquid jet injectors. Nat. Rev. Drug Discov..

[18] Arora A (2007). Needle-free delivery of macromolecules across the skin by nanoliter-volume pulsed microjets. Proc. Natl Acad. Sci. USA.

[19] Taberner A (2012). Needle-free jet injection using real-time controlled linear Lorentz-force actuators. Med. Eng. Phys. Sci..

[20] Lingeman JE, McAteer JA, Gnessin E, Evan AP (2009). Shock wave lithotripsy: advances in technology and technique. Nat. Rev. Urol..

[21] Ito K, Fukumoto Y, Shimokawa H (2009). Extracorporeal shock wave therapy as a new and non-invasive angiogenic strategy. Tohoku J. Exp. Med..

[22] van der Jagt OP (2009). Unfocused extracorporeal shock wave therapy as potential treatment for osteoporosis. J. Orthop. Res..

[23] Rathod, V. T. Optimization of a diaphragm for a micro-shock tube-based drug delivery method. *Bioengineering* **4**, 24 (2017). 10.3390/bioengineering4010024 PMC5590438 28952503

[24] Tagawa Y (2013). Needle-free injection into skin and soft matter with highly focused microjets. Lab A Chip.

[25] Jagadeesh G (2011). Needleless vaccine delivery using micro-shock waves.. Clin. Vaccine. Immunol..

[26] Yang GL (2009). Study on caculation method for resisitance of double V-shaped semiconductor bridge. Initiators Pyrotechics.

[27] Yu QX, Li MY (2018). Copper azide fabricated by nanoporous copper precursor with proper density. Appl. Surf. Sci..

