# Supplementary material for: A microexplosive shockwave-based drug delivery microsystem for treating hard-to-reach areas in the human body
Source: Microsyst Nanoeng. 2022 Sep 23;8:106. doi: 10.1038/s41378-022-00441-8 (PMC9508092; doi:10.1038/s41378-022-00441-8)
Supplement: Supplementary file 2 — Supplementary figure [file 41378_2022_441_MOESM2_ESM.docx]

**
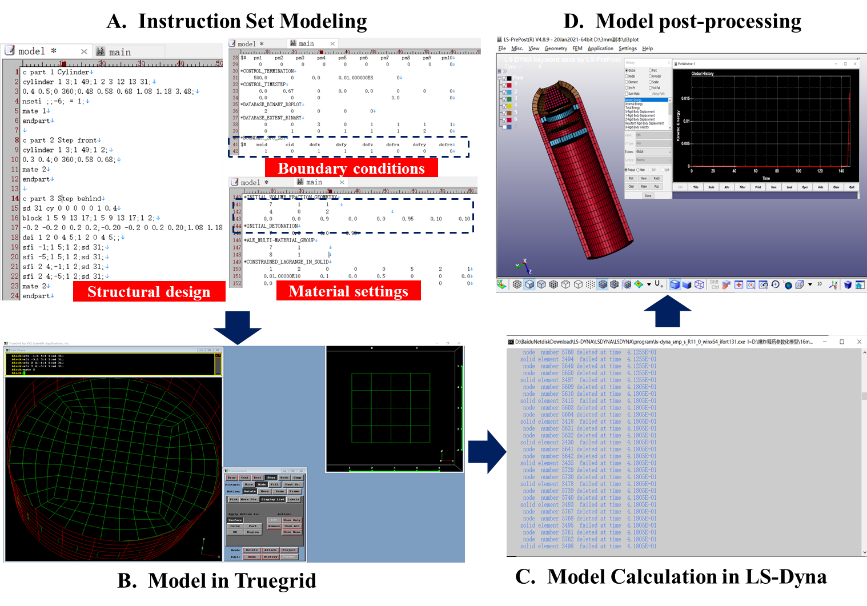
**

**Figure. S1**


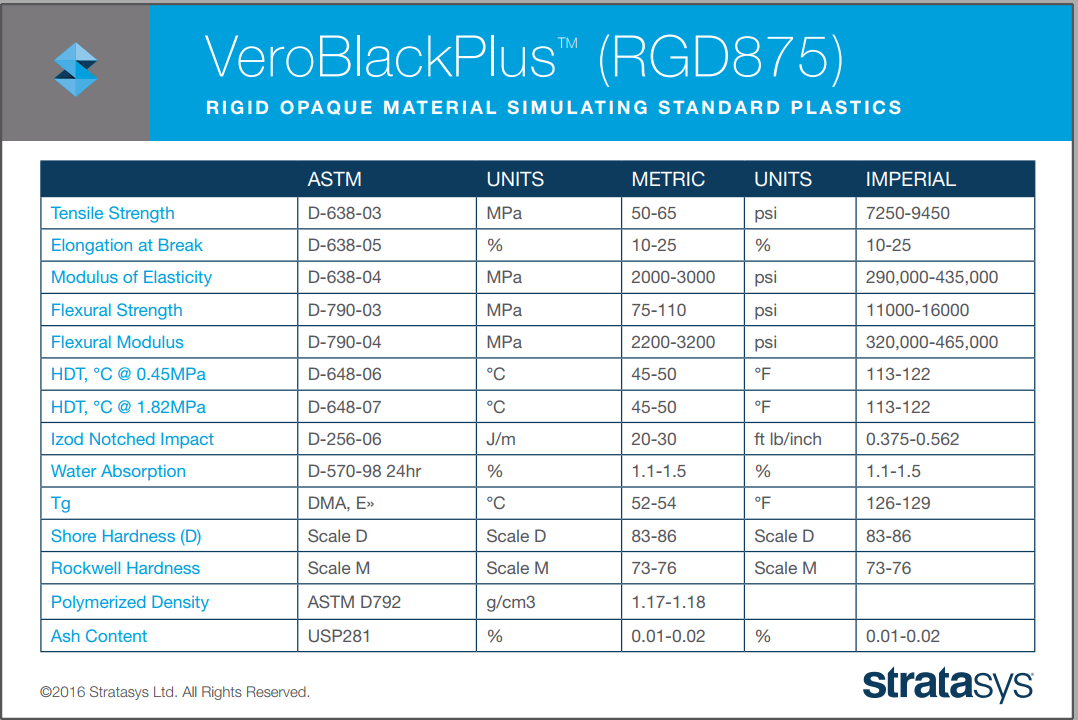


**Figure. S2**


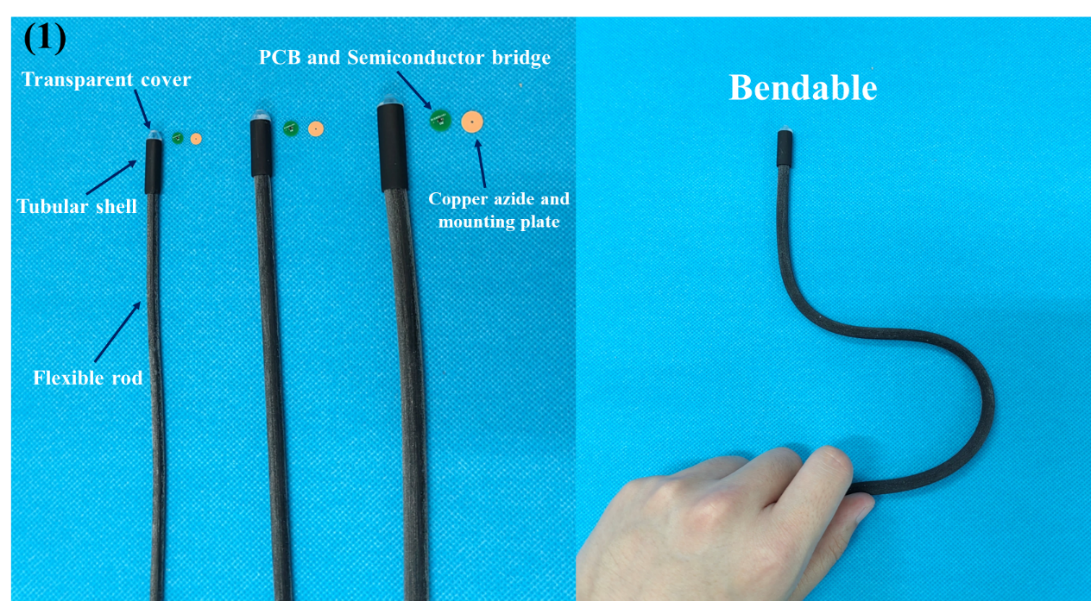

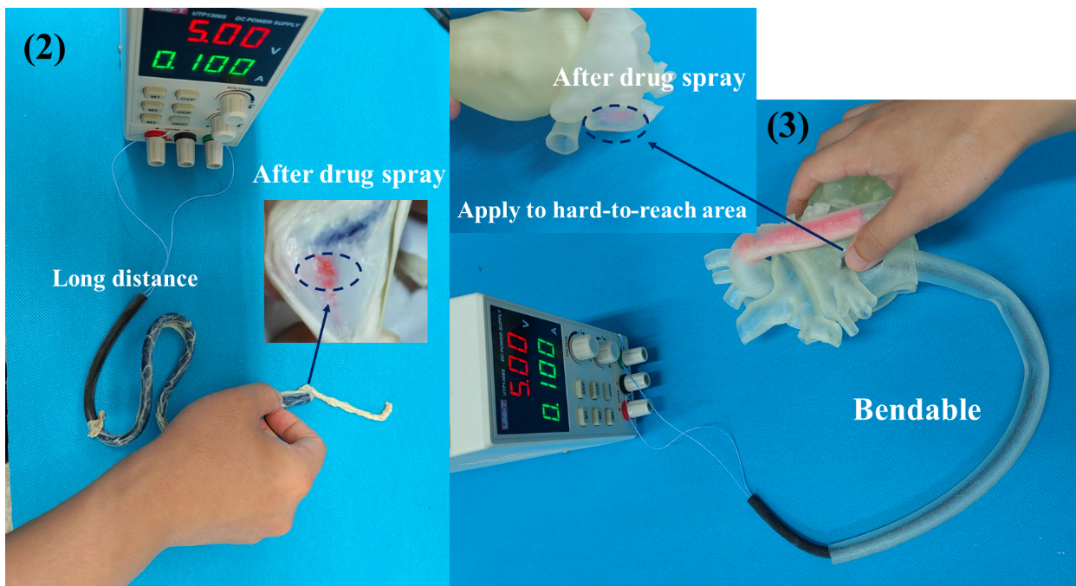


**Figure. S3**


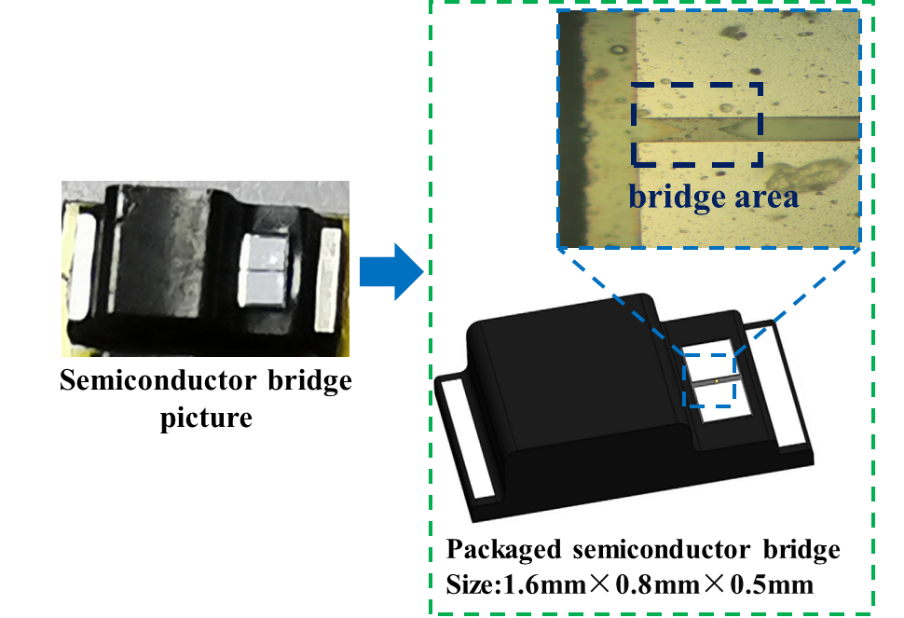


**Figure. S4**

**Table S1. Comparison of devices from different studies and the device of this work**

|  | Sahab Babaee | Andrew Taberner | Rathod | **This work** |
| --- | --- | --- | --- | --- |
| Material and size | Hard silicone, Diameter 12.5 mm | Lorentz-force motor, cRIO controller | Polymer tube, Aluminum film | **Flexible material, Diameter 4 mm~1 cm** |
| Working principle | Microneedle penetration to release drug | High pressure jet of liquid | Explosive spray | **Explosive spray** |
| Application area | in vivo (gastrointestinal tract) | in vitro | in vitro (skin) | **Hard-to-reach areas in vivo** |
| Drug release speed | Slow (a few seconds) | Fast (a few milliseconds) | Fast (a few milliseconds) | **very fast (a few microseconds)** |
| Energy consumption | High pressure drive | 4 kW high power drive | Over 12 V | **Low voltage 5 V drive** |
| Biosafety | Medium | Medium | Poor | **Very good** |
